# The Selective LAT1 Inhibitor JPH203 Enhances Mitochondrial Metabolism and Content in Insulin-Sensitive and Insulin-Resistant C2C12 Myotubes

**DOI:** 10.3390/metabo13060766

**Published:** 2023-06-19

**Authors:** Caroline N. Rivera, Carly E. Smith, Lillian V. Draper, Gabriela E. Ochoa, Rachel M. Watne, Andrew J. Wommack, Roger A. Vaughan

**Affiliations:** 1 Department of Exercise Science, High Point University, High Point, NC 27268, USA; crivera@highpoint.edu (C.N.R.); csmith19@highpoint.edu (C.E.S.); ldraper@highpoint.edu (L.V.D.); gochoa@highpoint.edu (G.E.O.); 2 Department of Chemistry, High Point University, High Point, NC 27268, USA; rwatne@highpoint.edu (R.M.W.); awommack@highpoint.edu (A.J.W.)

**Keywords:** leucine, isoleucine, valine, pAkt/Akt, skeletal muscle, insulin resistance, diabetes

## Abstract

Population data have shown an association between higher circulating branched-chain amino acids (BCAA) and the severity of insulin resistance in people with diabetes. While several studies have assessed BCAA metabolism as a potential target for regulation, less attention has been paid to the role of L-type amino acid transporter 1 (LAT1), the primary transporter of BCAA in skeletal muscle. The aim of this study was to assess the impact of JPH203 (JPH), a LAT1 inhibitor, on myotube metabolism in both insulin-sensitive and insulin-resistant myotubes. C2C12 myotubes were treated with or without 1 μM or 2 μM JPH for 24 h with or without insulin resistance. Western blot and qRT-PCR were used to assess protein content and gene expression, respectively. Mitochondrial and glycolytic metabolism were measured via Seahorse Assay, and fluorescent staining was used to measure mitochondrial content. BCAA media content was quantified using liquid chromatography–mass spectrometry. JPH at 1 μM (but not 2 μM) increased mitochondrial metabolism and content without inducing changes in mRNA expression of transcripts associated with mitochondrial biogenesis or mitochondrial dynamics. Along with increased mitochondrial function, 1μM treatment also reduced extracellular leucine and valine. JPH at 2 μM reduced pAkt signaling and increased extracellular accumulation of isoleucine without inducing changes in BCAA metabolic genes. Collectively, JPH may increase mitochondrial function independent of the mitochondrial biogenic transcription pathway; however, high doses may reduce insulin signaling.

## 1. Introduction

Branched-chain amino acids (BCAA) are required in the human diet, though population data has linked elevated circulating BCAA with severity of insulin resistance [1,2,3,4,5]. Constitutive mechanistic target of rapamycin complex 1 (mTORC1) activation leading to desensitized insulin receptor substrate (IRS) [4], excess acyl-carnitine accumulation [5,6], 3-hydroxyisobutyrate-mediated lipid accumulation [7,8,9,10], and inhibition of mitochondrial function are some of the suggested mechanisms by which BCAA may worsen insulin resistance [4,5]. Recently, several reports have demonstrated that the stimulation of BCAA metabolism may improve several of these metabolic phenotypes including insulin resistance. For example, 4-phenylbutyrate (PBA), a known scavenger of ammonia [11], has been shown to restore BCAA-mediated suppression of insulin signaling in cultured myotubes [12]. PBA has also been shown to reduce mechanistic target of rapamycin (mTOR) signaling and protein synthesis [13], ER stress [14], and palmitate-induced inflammation [15]. Thus, the exact mechanisms by which PBA functions to resolve insulin resistance are still unclear. However, administration of PBA daily for 2 weeks improved BCAA metabolism and insulin resistance in humans with type 2 diabetes, as well as mitochondrial function in permeabilized skeletal muscle [16].

While agents such as PBA are being explored for their ability to combat BCAA accumulation and insulin resistance, less attention has been paid to the manipulation of L-type amino acid transporter 1 (LAT1), the predominant transporter of BCAA in skeletal muscle. JPH203 (JPH) is a LAT1 inhibitor which may have applications for treating certain cancers [17,18]. JPH appears to reduce mTORC1 signaling in HepG2 cells and reduce liver triglycerides in high-fat-fed C57BL/6J mice [19]. JPH has been shown to depress mitochondrial and glycolytic metabolism and related gene expression in Th17 cells [20]. Yet, although JPH and LAT1 inhibition seem to possess promising therapeutic potential, several experiments have previously revealed the dispensable nature of LAT1 in the maintenance of intracellular BCAA levels, likely compensated for by other transport proteins [21,22,23]. For example, LAT1-knockout mice exhibit normal leucine levels with simultaneously increased LAT2 mRNA expression (*Slc7a8*) [23]. Moreover, myocyte LAT1 abundance does not seem to be dependent on cell culture media leucine content [24], nor is skeletal muscle LAT1 content dependent on dietary protein intake [25]. 

Though documented for potential efficacy in the treatment of select cancers [17], to date, inhibition of and therapies that target LAT1 (such as JPH) are currently underexplored in the pathology of insulin resistance in skeletal muscle. It seems possible that LAT1 inhibition may alter BCAA behavior in skeletal muscle, which may have implications for metabolic diseases such as insulin resistance. However, the dispensability of LAT1 in the maintenance of intracellular BCAA content suggests that more than LAT1 inhibition may be required to elicit any effect. The purpose of the present report is to evaluate the effect of JPH at physiologically attainable concentrations on myotube metabolism in both insulin-sensitive and insulin-resistant myotubes.

## 2. Experimental Methods

### 2.1. Cell Culture

C2C12 mouse myoblasts, catalog CRL-1772 from ATCC (Manassas, VA, USA), were cultured in Dulbecco’s Modified Eagle’s Medium (DMEM) containing 4500 mg/L glucose and supplemented with a 20% heat-inactivated fetal bovine serum (FBS), a 100 U/mL penicillin and a 100 µg/mL streptomycin in a humidified 5% CO_2_ atmosphere at 37 °C. Cells were grown to confluency with growth media changed every 2 to 3 days (using cell passages <20 for all experiments). Differentiation was accomplished by replacing growth media with DMEM supplemented with a 2% horse serum, a 100 U/mL penicillin and a 100 µg/mL streptomycin for 6 days. Similar to previous experiments [26,27,28,29,30], insulin resistance was accomplished by the addition of insulin at 100 nM for the final 3 days of differentiation, which significantly reduces insulin signaling without altering cell differentiation status [29,30]. Cells were then treated with JPH203 dissolved in DMSO and a final concentration of 1 µM or 2 µM (with 0.1% or 0.2% DMSO (*v/v*) used as vehicle or control) or control for 24 h. These concentrations were chosen based on initial data demonstrating human subjects with tumors administered 12–85 mg/m^2^ displayed JPH203 blood concentrations that approximated 0.4–3.8 µM, with most concentrations (25, 40, and 60 mg/m^2^) falling between the 1 and 2 µM after a single dose. Thus, 1–2 µM can be considered as achievable and relevant physiological concentrations in humans [18]. Moreover, in vitro experiments demonstrated that 1 µM using a similar vehicle delivery condition is sufficient to reduce leucine uptake [17]. Collectively, we reasoned that the 1–2 µM concentrations were most appropriate to represent physiologically attainable levels that could also conceivably illicit a substantial inhibitory effect on LAT1-mediated BCAA transport. 

### 2.2. Cell Viability

To assess cell viability, cells were treated as described above, and media were then replaced with media containing a 10% WST-1 reagent (*v/v*) from Cayman Chemical (Ann Arbor, MI, USA). Absorbance at 450 nm was measured temporally for 90 min. Main effect for JPH at 2 µM (but not 1 µM) was observed during experiments with 2 µM (Figure S1); however, no group differences were observed during pairwise comparisons for any of the experimental conditions (Figure S1). 

### 2.3. Quantitative Real-Time Polymerase Chain Reaction (qRT-PCR)

Cells were differentiated and treated as described above. Total mRNA was extracted using the Trizol method and quantified (via NanoDrop from Thermo Fisher, Wilmington, DE, USA), and cDNA was synthesized using the iScript cDNA Synthesis Kit from Bio-Rad (Hercules, CA, USA) according to manufacturer’s instructions. PCR primers were synthesized by Integrated DNA Technologies (Coralville, IA, USA) (Table S1). Amplification of target genes was normalized to the housekeeping gene, the TATA-binding protein (*Tbp*), which did not differ between groups (*Tbp*, shown in Figure S2). qRT-PCR reactions were performed using the CFX Connect System from Bio-Rad (Hercules, CA, USA). SYBR Green-based PCR was performed using final primer concentrations at 3.75 µM in a total volume of 10 µL per well. The following cycling parameters were used: 95 °C for 3 min followed by 40 cycles of 95 °C for 15 s, and 60 °C for 30 s. qRT-PCR reactions were performed using *n* = 3 per treatment condition from two independent experiments with *n* = 6 for the final analysis. Relative quantification was determined via the ΔΔCt method. 

### 2.4. Immunoblotting 

To investigate the effect of JPH with and without insulin resistance on insulin signaling, the cells were differentiated and treated as described above followed by serum-free media stimulation with (100 nM) and without insulin for 30 min. Whole cell lysates were then prepared by harvesting the cells on ice in a RIPA buffer supplemented with protease inhibitor, followed by incubation on ice for 60 min. Insoluble material was removed, and protein concentrations were determined by the Bradford assay. Total protein (50 μg per sample) was size-separated by 10% sodium dodecyl sulfate polyacrylamide gel electrophoresis (SDS-PAGE) and electro-transferred to PVDF membranes. After blocking in TBST-5% non-fat milk powder for 1 h, membranes were probed at 4 °C overnight with the pAkt (sc-7985-R) and Akt (sc-8312) polyclonal antibodies from Santa Cruz Biotechnologies (Santa Cruz, CA, USA) at a dilution of 1:1000 in TBST-5% non-fat milk powder. Bound antibodies were detected by horseradish peroxidase-conjugated secondary antibodies from AbCam (Cambridge, MA, USA) at a dilution of 1:5000 in TBST-5% non-fat milk powder for 1 h at room temperature while shaking. Protein signal intensities were determined by chemiluminescence using the Clarity Western ECL substrate kit from Bio-Rad (Hercules, CA, USA) and imaged using the ChemiDoc Touch from Bio-Rad (Hercules, CA, USA). Relative signal intensities were quantified using Image Lab from Bio-Rad (Hercules, CA, USA). Blots were performed using 3 replicates per condition performed across 2 independent experiments with *n* = 6 for the final analysis. Each target was also measured in duplicate for each sample and the average used in the final analyses. Molecular weights for all targets were verified against sizes suggested by product brochures.

### 2.5. Seahorse Metabolic Assays

Cells were seeded into Seahorse XFe96 culture plates, differentiated, and treated as described above. Media were then replaced with XF Assay Media obtained from Agilent Technologies (Santa Clara, CA, USA) containing glucose at 25 mM, pyruvate at 1 mM, and glutamine at 2 mM. Following incubation, baseline measurements of oxygen consumption rate (OCR) and extracellular acidification rate (ECAR) were recorded as indicators of basal oxidative metabolism and glycolytic metabolism, respectively. Following basal measurements, each well was infused with oligomycin (an inhibitor of ATP synthase) at a final concentration of 2 μM to induce maximal glycolytic metabolism. Cells were then exposed to carbonyl cyanide p-[trifluoromethoxy]-phenyl-hydrazone (FCCP) at 2 μM to uncouple electron transport and induce peak OCR. Maximal respiration measurements were followed by the injection of rotenone at 1 μM to reveal non-mitochondrial respiration. Basal and peak oxidative metabolisms were normalized to non-mitochondrial OCR from each respective well. The Seahorse XFe96 Analyzer was run using a 6 min cyclic protocol command (mix for 3 min and measure for 3 min). MitoStress assays included *n* = 23 per group repeated with two independent experiments for *n* = 46 per group for the final analysis. States of mitochondrial metabolism were calculated by subtracting non-mitochondrial respiration from basal or FCCP-induced peak mitochondrial oxygen consumption. Wells with negative OCR values or no response to injection were removed from the final analysis.

### 2.6. Fluorescent Staining and Microscopy

Immediately following the Seahorse metabolic assay described above, cells were fixed using 3.7% formaldehyde at 37 °C with a 5% CO_2_ atmosphere. The fixing agent was then removed, and cells were stained with DAPI at 0.5 µM in PBS and fluorescence was measured at 360/460 nM (Figure S3). Cells were then stained with a 100 µM nonyl acridine orange (NAO) (Fremont, CA, USA) in PBS and incubated in the dark at room temperature for 10 min. Fluorescence was then measured using 485/525 nm excitation/emission. Neutral lipid content was measured using Nile Red staining at 10 µM PBS with 1% DMSO (*v/v*) using 530/645 nm excitation/emission. All fluorescent measurements were performed in triplicate and the average (less background) analyzed with *n* = 23 per group repeated with two independent experiments with *n* = 46 per group for the final analyses. Following fluorescent quantification, cells were imaged via the 10× objective using the Motic AE31E inverted microscope and Moticam Pro 252B (Causeway Bay, Hong Kong). 

### 2.7. Liquid Chromatography–Mass Spectrometry (LC–MS)

Similar to previous experiments [31], media BCAA content was assessed to determine extracellular BCAA content/accumulation. Chromatographic separation and quantification of leucine, isoleucine, and valine was performed using a Shimadzu Nexera UHPLC system equipped with a Phenomenex Kinetex C18 100 Å column (100 × 3 mm, 2.6 µm) kept at a temperature of 30 °C connected to a Shimadzu LCMS-8045 triple quadrupole mass spectrometer (Shimadzu, Kyoto, Japan) fitted with a DUIS ion source. The source used nebulizer gas 2.0 L/min, drying gas 10.0 L/min, desolvation line (DL) temperature 200 °C and heat block temperature 200 °C, with CID gas 230 kPa. The mobile phases of A (water with 0.1% formic acid) and B (methanol 0.1% formic acid) were used at a flow rate of 0.4 mL/min for the following gradient method: 0 min, 20% B; 1.5 min, 20% B; 1.7 min, 40% B; 3.5 min, 40% B; 5 min, 65% B; 8 min, 65% B; followed by 4 min 20% B for column equilibration. The injection volume was maintained at 1 µL. This afforded reproducible retention time values for valine (1.112 min), isoleucine (1.356 min), and leucine (1.425 min).

Shimadzu LabSolution software version 5.97 was used to acquire and process the data. The fragmentation for each BCAA was optimized using an MRM set to positive mode for valine (118.1 to 72.2 *m*/*z*, Q1 −23.0 V, CE −12.0 V, and Q3 −20.0 V), isoleucine (132.0 to 69.2 *m*/*z*, Q1 −10.0 V, CE −19.0 V, and Q3 −11.0 V), and leucine (132.1 to 43.2 *m*/*z*, Q1 −10.0 V, CE −26.0 V, and Q3 −18.0 V), with a dwell time of 100 ms.

A stock solution containing all BCAAs at a concentration of 8.0 mM was obtained by dissolving each amino acid in a water/methanol solution (50:50, *v*/*v*) and kept at 4 °C. Further dilutions with water/methanol were performed to assemble a calibration curve ranging from 3.125 to 100.0 µM. Experiments were performed using 3 replicates per group for each of 2 independent experiments with *n* = 6 for each group in the final analyses.

### 2.8. Statistical Analyses

Data are presented as dot plots with group means, or as group mean ± SE. Data were analyzed with two-way ANOVA with subsequent one-way ANOVA with Bonferroni’s correction for pairwise group differences. Values of *p* ≤ 0.05 were used to identify significant differences between groups.

## 3. Results

### 3.1. JPH at High Levels Reduces Insulin Sensitivity in Previously Insulin-Resistant Cells

**Figure 1:**
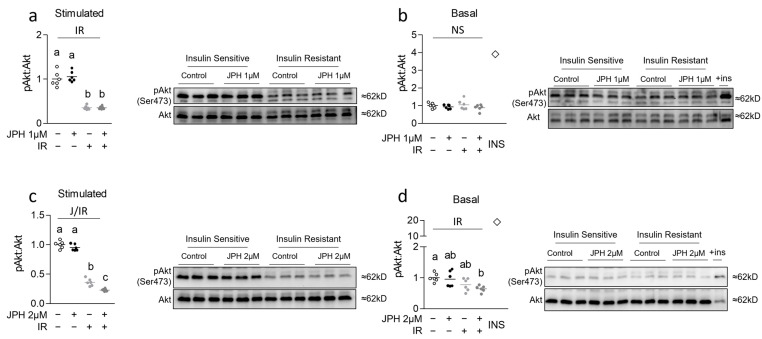
Effect of JPH with and without insulin resistance on insulin signaling. (**a**,**b**) Effect of JPH-203 (JPH) at 1 µM for 24 h with and without insulin resistance (IR) induced via supplementation with 100 nM insulin for 3 days on insulin signaling (pAkt:Akt) following either (**a**) insulin stimulation for 30 min or (**b**) media without insulin stimulation (basal) for 30 min (with positive insulin-stimulated control at far right indicated by a diamond for reference (+ins or INS)). (**c**,**d**) Effect of JPH at 2 µM for 24 h with and without IR on pAkt:Akt following either (**c**) insulin stimulation for 30 min or (**d**) media without insulin stimulation (basal) for 30 min (with positive insulin-stimulated control at far right for reference). Notes: pAkt expression was analyzed with two-way ANOVA and subsequent one-way ANOVA with Bonferroni’s correction for multiple comparisons. Dissimilar letters within each graph and above each group indicate *p* ≤ 0.05 between groups, while J, IR, and I represent main and interaction effects for two-way ANOVA (with NS indicating no significant effect). Experiments were performed using *n* = 3 per group across 2 independent experiments with *n* = 6 for the final analysis with each target measured in duplicate.

We began our investigation by assessing the effect of JPH both with and without insulin resistance on both basal and insulin-stimulated pAkt expression. Insulin resistance was observed in all insulin-resistant groups as evidenced by reduced pAkt expression following insulin stimulation (Figure 1a,c). At 1 µM, JPH had no effect on basal or insulin-stimulated pAkt expression (Figure 1a,b, respectively); however, JPH at 2 µM for 24 h significantly reduced insulin-stimulated and basal pAkt expression in insulin-resistant but not insulin-sensitive cells (Figure 1c,d, respectively). 

### 3.2. JPH Improves Mitochondrial Function without Altering Mitochondrial Biogenic Signaling 

**Figure 2:**
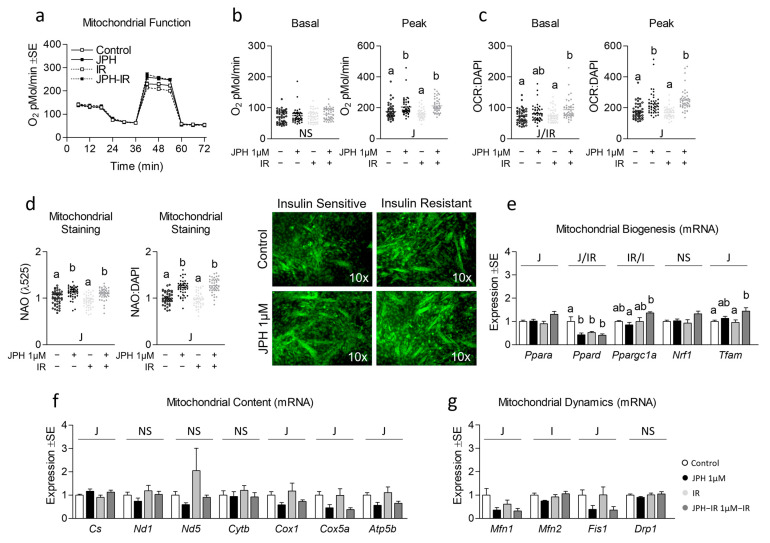
Effect of JPH both with and without insulin resistance on mitochondrial biogenic signaling, content, and function. (**a**) Time course of the effect of JPH-203 (JPH) at 1 µM for 24 h with and without insulin resistance (IR) on mitochondrial metabolism. (**b**,**c**) Effect of JPH with and without IR on basal (left) and peak (right) mitochondrial metabolism both without (**b**) and with (**c**) normalization to cell nuclei content (presented in Figure S3). (**d**) Mitochondrial content of cells described in “a” indicated by NAO staining without (left) and with normalization to cell nuclei content (presented in Figure S3). (**e**) Effect of JPH with and without IR on mRNA expression of mitochondrial biogenesis including peroxisome proliferator-activated receptor-alpha (*Ppara*), peroxisome proliferator-activated receptor-delta (*Ppard*), peroxisome proliferator-activated receptor-gamma coactivator-1alpha (*Ppargc1a*), nuclear respiratory factor 1 (*Nrf1*), and mitochondrial transcription factor A (*Tfam*). (**f**) Effect of JPH with and without IR on mRNA expression of mitochondrial content including citrate synthase (*Cs*), cytochrome c oxidase 1 (*Cox1*), NADH dehydrogenase 1 (*Nd1*), NADH dehydrogenase 5 (*Nd5*), cytochrome b (*Cytb*), cytochrome c oxidase subunit 5a (*Cox5a*), and ATP synthase subunit 5b (*Atp5b*). (**g**) Effect of JPH with and without IR on mRNA expression of mitochondrial dynamics including mitofusin 1 (*Mtfn1*), mitofusin 2 (*Mfn2*), mitochondrial fission protein 1 (*Fis1*), and Dynamin-related protein 1 (*Drp1*). Notes: Two-way ANOVA and one-way ANOVA with Bonferroni’s correction for multiple comparisons were used to assess differences in metabolism, mitochondrial content, and gene expression following 24 h treatment. Dissimilar letters within each graph and above each group indicate *p* ≤ 0.05 between groups, while J, IR, and I represent main and interaction effects for two-way ANOVA (with NS indicating no significant effect). States of mitochondrial metabolism were calculated by subtracting non-mitochondrial respiration from basal or FCCP-induced peak oxygen consumption. Metabolic measurements were performed using *n* = 23 individual replicates per treatment condition and were repeated across 2 independent experiments with *n* = 46 per group in the final analyses. No wells responded with negative raw values. Mitochondrial staining was performed using *n* = 23 individual replicates per treatment condition and were repeated across 2 independent experiments with *n* = 46 per group in the final analyses using the average of 3 measurements per experiment (less background). Images in “d” of representative individual myotubes were taken using the 10× objective. Target gene expression was normalized to average TATA-binding protein (*Tbp*) using 3 replicates per group across 2 independent experiments with *n* = 6 for the final analysis.

**Figure 3:**
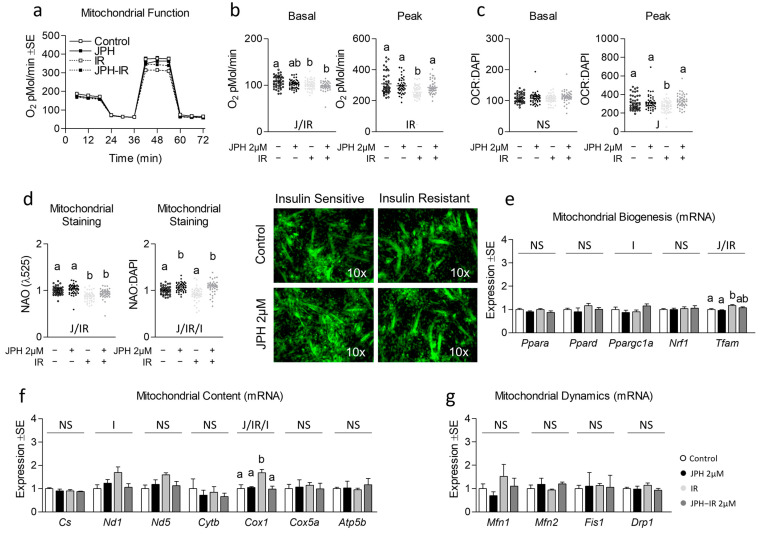
Effect of high-dose JPH both with and without insulin resistance on mitochondrial biogenic signaling, content, and function. (**a**) Time course of the effect of JPH-203 (JPH) at 2 µM for 24 h with and without insulin resistance (IR) on mitochondrial metabolism. (**b**,**c**) Effect of JPH with and without IR on basal (left) and peak (right) mitochondrial metabolism both without (**b**) and with (**c**) normalization to cell nuclei content (presented in Figure S3). (**d**) Mitochondrial content of cells described in “a” indicated by NAO staining without (left) and with normalization to cell nuclei content (presented in Figure S3). (**e**) Effect of JPH with and without IR on mRNA expression of mitochondrial biogenesis including peroxisome proliferator-activated receptor-alpha (*Ppara*), peroxisome proliferator-activated receptor-delta (*Ppard*), peroxisome proliferator-activated receptor-gamma coactivator-1alpha (*Ppargc1a*), nuclear respiratory factor 1 (*Nrf1*), and mitochondrial transcription factor A (*Tfam*). (**f**) Effect of JPH with and without IR on mRNA expression of mitochondrial content including citrate synthase (*Cs*), cytochrome c oxidase 1 (*Cox1*), NADH dehydrogenase 1 (*Nd1*), NADH dehydrogenase 5 (*Nd5*), cytochrome b (*Cytb*), cytochrome c oxidase subunit 5a (*Cox5a*), and ATP synthase subunit 5b (*Atp5b*). (**g**) Effect of JPH with and without IR on mRNA expression of mitochondrial dynamics including mitofusin 1 (*Mtfn1*), mitofusin 2 (*Mfn2*), mitochondrial fission protein 1 (*Fis1*), and Dynamin-related protein 1 (*Drp1*). Notes: Two-way ANOVA and one-way ANOVA with Bonferroni’s correction for multiple comparisons were used to assess differences in metabolism, mitochondrial content, and gene expression following 24 h treatment. Dissimilar letters within each graph and above each group indicate *p* ≤ 0.05 between groups, while J, IR, and I represent main and interaction effects for two-way ANOVA (with NS indicating no significant effect). States of mitochondrial metabolism were calculated by subtracting non-mitochondrial respiration from basal or FCCP-induced peak oxygen consumption. Metabolic measurements were performed using *n* = 23 individual replicates per treatment condition and were repeated across 2 independent experiments with *n* = 46 per group in the final analyses. No wells responded with negative raw values. Mitochondrial staining was performed using *n* = 23 individual replicates per treatment condition and were repeated across 2 independent experiments with *n* = 46 per group in the final analyses using the average of 3 measurements per experiment (less background). Images in “d” of representative individual myotubes were taken using the 10× objective. Target gene expression was normalized to average TATA-binding protein (*Tbp*) using 3 replicates per group across 2 independent experiments with *n* = 6 for the final analysis.

Next, because mitochondrial function is often altered during insulin resistance, we assessed the effect of JPH on mitochondrial function in both insulin-sensitive and insulin-resistant cells (Figure 2a). JPH at 1 µM significantly increased peak mitochondrial function in both insulin-sensitive and insulin-resistant myotubes both before and after controlling for nuclear content (Figure 2b,c, respectively). Consistent with these observations, JPH at 1 µM also significantly increased mitochondrial content in both insulin-sensitive and insulin-resistant myotubes (Figure 2d), which ratio-metrically only accounted for a portion of the increase observed in peak mitochondrial function. Surprisingly, however, JPH at 1 µM did not result in an induction of mitochondrial biogenesis, content, or mitochondrial dynamics at the mRNA level (Figure 2e–g, respectively). At higher levels, JPH did not elicit the same effects. Specifically, JPH at 2 µM did not increase peak mitochondrial metabolism in insulin-sensitive or insulin-resistant cells (Figure 3a–c). However, after controlling for nuclear content, JPH did increase mitochondrial staining in both levels of insulin sensitivity (Figure 3d). In addition, like gene expression following treatment with JPH at 1 µM, JPH at 2 µM did not significantly alter expressional indicators of mitochondrial biogenesis, mitochondrial content, or mitochondrial dynamics (Figure 3e–g, respectively). In addition to mitochondrial metabolism and related gene expression, we also assessed the effect of both JPH doses on glycolytic metabolism, lipid content, and related gene expression for each. In general, JPH reduced both glycolytic metabolism and lipid content, though after correcting for nuclei content, these differences were mostly insignificant compared to respective controls (Figures S4 and S5, respectively). Similarly, glycolytic and lipogenic gene expression was largely unaltered by JPH treatment (Figures S4 and S5, respectively).

### 3.3. Effect of JPH on Extracellular BCAA Content with and without Insulin Resistance

**Figure 4:**
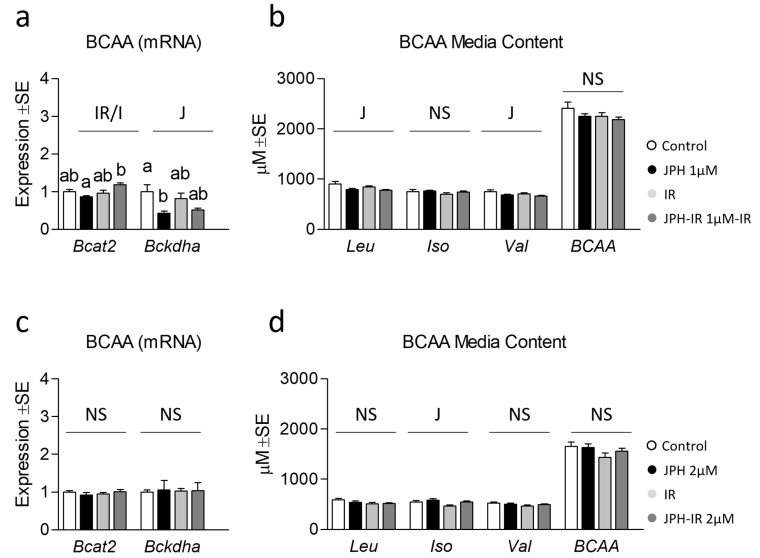
Effect of JPH with and without insulin resistance on BCAA catabolic enzymes and media BCAA content. (**a**) Effect of JPH-203 (JPH) at 1 µM for 24 h with and without insulin resistance (IR) on mRNA expression of branched-chain amino acid transaminase 2 (*Bcat2*) and branched-chain alpha-keto acid dehydrogenase a (*Bckdha*). (**b**) Media BCAA content from cells following treatment as described in “a” for 24 h. (**c**) Effect of JPH at 2 µM for 24 h with and without IR on lipogenic mRNA expression *Bcat2* and *Bckdha*. (**d**) Media BCAA content from cells following treatment as described in “c” for 24 h. Notes: Two-way ANOVA and one-way ANOVA with Bonferroni’s correction for multiple comparisons were used to assess differences in media BCAA content and gene expression. Dissimilar letters within each graph and above each group indicate *p* ≤ 0.05 between groups, while J, IR, and I represent main and interaction effects for two-way ANOVA (with NS indicating no significant effect). Target gene expression was normalized to average TATA-binding protein (*Tbp*) using 3 replicates per group across 2 independent experiments with *n* = 6 for the final analysis. Media content was analyzed using 3 replicates per group across 2 independent experiments with *n* = 6 for the final analysis.

Lastly, because JPH is a selective LAT1 inhibitor and because we observed increased mitochondrial function and/or content following treatment, we next assessed indicators of BCAA catabolism and extracellular BCAA content following JPH treatment. Though JPH at 1 µM had no significant effect on *Bcat2* expression, a main effect and significant reduction of *Bckdha* expression by JPH was observed following a 1 µM treatment (Figure 4a). Perplexingly, we observed subtle reductions in leucine and valine, but not isoleucine or BCAA media content in cells treated with JPH at 1 µM (Figure 4b). We speculate that increased BCAA disposal may have been supported by heightened mitochondrial respiration associated with JPH at 1 µM shown in Figure 2. For JPH at 2 µM, no effect was observed on either *Bcat2* or *Bckdha* (Figure 4c); however, a significant increase in isoleucine was observed across cells treated with JPH at 2 µM (Figure 4d), which is more consistent with the inhibitor effect of JPH on BCAA uptake. Interestingly, neither dose altered total BCAA accumulation, which might be explained by the redundancy of BCAA transport mechanisms. 

## 4. Discussion

Previous experiments have demonstrated a dispensable nature of LAT1, which appears to be compensated for by other transports in the maintenance of tissue BCAA content [21,22,23]. For example, gastrocnemius of LAT1-knockout mice exhibits either elevated (fasted) or normal (fed) leucine content versus wild-type littermates, along with increased LAT2 mRNA expression (*Slc7a8*) [23]. Additionally, it appears LAT1 that abundance is dependent on neither media leucine content in myocytes [24] nor dietary protein intake within skeletal muscle [25]. Yet, despite this dispensable nature, JPH203 remains a potential therapeutic option in the treatment of metabolic diseases such as cancer [17]. 

One perplexing finding within our study was the observation of rescued mitochondrial function in 2 µM JPH-treated insulin-resistant cells versus insulin-resistant only (Figure 3b,c), yet decreased insulin signaling in JPH-treated cells following insulin stimulation (Figure 1c). This finding is in contrast to our initial hypothesis and seems paradoxical given increased mitochondrial function is typically associated with improved insulin sensitivity. Although speculative, it could be that the seemingly inconsistent findings are explained by the interplay between mTORC1 and Akt signaling. Inhibitors of mTORC1 such as rapamycin have shown biphasic effects on insulin signaling [32], which is associated with reduced mitochondrial function (but not content). Indeed, increased duration of mTORC1 inhibition via rapamycin was associated with progressively reduced mitochondrial function [32]. Similar observations were demonstrated by Cunningham et al., who convincingly showed the importance of mTORC1 in the regulation of mitochondrial content as well as function [33]. Previous observations have linked JPH to reduced mTORC1 and Akt activation [34,35], although it should be noted that many of the mechanistic observations of JPH have been shown using cancer cell culture models, which may not be representative of normal muscle insulin signaling. It could be that JPH disrupts the established link between mTORC1 activation and insulin signaling via amino acids [36], which could also explain, in part, some of the antineoplastic effects of JPH (speculation supported by limited in vitro observations [19]). Further speculation might lead one to hypothesize that JPH-mediated suppression of mTORC1 signaling might occur with simultaneously upregulated AMPK (a known negative regulator of mTORC1 in many circumstances [37]). Conversely, however, we did not consistently observe upregulated transcripts of mitochondrial biogenesis or other pathways one might expect to find associated with heightened AMPK activation. Thus, much of the mechanistic detail of how JPH functions remains unknown, and a major limitation of our study was the lack of protein expression measurements during acute timepoints. Similarly, while we assessed numerous mRNA transcripts associated with cell metabolism, we did not assess the protein expression of these targets. Additionally, prior in vitro models have used concentrations that exceed those used in the present report manyfold [34]. Importantly, we only observed a significant suppression of insulin-mediated pAkt activation in insulin-resistant (but not -sensitive) cells treated with JPH at 2 µM (higher physiologically attainable) but not 1 µM (lower physiologically attainable) [18], suggesting that these findings are also concentration-dependent. 

Despite the negative regulation of insulin resistance, we observed heightened mitochondrial function and content in cells treated with JPH at lower doses. It was surprising to us that these metabolic adaptations occurred independent of upregulation of the mitochondrial biogenic transcriptional program; however, it could be that JPH-mediated changes in mitochondrial content/function were mediated via other targets that were not assessed in the current report or occurred prior to the 24 h data collection time point. Additionally, only subtle differences in glycolytic metabolism (Figure S4), lipid content (Figure S5), and respective related gene expression were observed. Given JPH is an inhibitor of LAT1, the predominant BCAA transporter, we also assessed indicators of BCAA metabolism. Interestingly, we observed only subtle effects of JPH on BCAA media content, which may further support the observation that LAT1 is dispensable in the maintenance of cellular BCAA content. Additionally, it could be that these results are dependent on other substrate availability (such as glucose). Moreover, we observed no effect of insulin resistance on BCAA media content, which seems at odds with prior observations by our group, which showed a substantial accumulation of extracellular BCAA in insulin-resistant groups [31]; however, an important difference in these experiments is the lack of recovery from excess insulin in the present report, which appears to be necessary for hyperinsulinemic-mediated accumulation of extracellular BCAA. In our previous observations, we utilized a 24 h recovery period (void of additional insulin) which may be necessary to reveal the accumulating effects of insulin resistance.

## 5. Conclusions

To date, only limited evidence has assessed the effect of JPH on cell metabolism, and those findings conflict with the observation in our report [20], though, admittedly, the differences in experimental models and context are substantial. With that in mind, it is worth highlighting several study limitations and considerations. First, although we used physiologically attainable levels of JPH [18], we cannot exclude the possibility that our treatment conditions were not optimized to reveal alterations in the measured transcript (especially provided only one time point was assessed). Moreover, while we assessed BCAA concentration in the media following treatment, we did not vary BCAA levels to determine whether activity of JPH is dependent upon BCAA abundance. Globally, it is also important to highlight the proof-of-concept nature of these observations. Though the C2C12 myotube model is among the most commonly used for experiments in skeletal muscle physiology, the translatability of these findings in vivo requires additional research. Furthermore, while we selected JPH concentrations that are physiologically relevant given past observations, it could be that the therapeutic range to elicit effects of JPH203 on metabolism are narrow (also warranting further investigation). Thus, these data should be interpreted with these limitations in mind. Despite these limitations, we feel our report provides substantial preliminary data on the effect of JPH on myotube metabolism and BCAA catabolism, which may have useful application for various metabolic diseases.

## Data Availability

The data that support the findings of this study are presented within the manuscript and are available from the corresponding author upon reasonable request.

## Supplementary Materials

The following supporting information can be downloaded at: https://www.mdpi.com/article/10.3390/metabo13060766/s1, Figure S1: Cell viability; Figure S2: qRT-PCR loading control; Figure S3: Nuclei content from Seahorse metabolic assays; Figure S4: Effect of JPH with and without insulin resistance on glycolytic metabolism and related gene expression; Figure S5: Effect of JPH with and without insulin resistance on lipogenesis and lipid content; Table S1: Summary of qRT-PCR primers from Integrated DNA Technologies (Coralville, IA, USA).
