# Supplementary material for: The Selective LAT1 Inhibitor JPH203 Enhances Mitochondrial Metabolism and Content in Insulin-Sensitive and Insulin-Resistant C2C12 Myotubes
Source: Metabolites. 2023 Jun 19;13(6):766. doi: 10.3390/metabo13060766 (PMC10303577; doi:10.3390/metabo13060766)
Supplement: Supplementary file 1 [file metabolites-13-00766-s001.zip › metabolites-2378113-supplementary.pdf]

The selective LAT1 inhibitor JPH203 enhances mitochondrial metabolism and content in insulin-sensitive and insulin-resistant C2C12 myotubes

Supplementary Materials

Caroline N. Rivera,[1] Carly E. Smith,[1] Lillian V. Draper,[1] Gabriela E. Ochoa,[1] Rachel M. Watne,[2] Andrew J. Wommack,[2] and Roger A. Vaughan[1]*

*Author Correspondence:
Email: rvaughan@highpoint.edu
Address: One University Parkway, High Point, NC 27262-3598
Phone: 336-841-9688
ORIC ID: 0000-0002-4593-8807

[1] Department of Exercise Science, High Point University, High Point, NC
[2] Department of Chemistry, High Point University, High Point, NC

Supplemental Tables and Legends

Table s1. Summary of qRT-PCR primers from Integrated DNA Technologies (Coralville, IA). Abbreviations: Beta actin (*Actb*), ATP synthase F1 (*Atp5b*), branched-chain aminotransferase 2 (*Bcat2*), branched-chain alpha-keto acid dehydrogenase (*Bckdha*), CCAAT/enhancer-binding protein alpha (*Cebpa*), cytochrome C oxidase Subunit 1 (*Cox1*), cytochrome C oxidase Subunit 5A (*Cox5a*), citrate synthase (*Cs*), cytochrome B (*cytb*), dynamin-related protein 1 (*Drp1*), fatty acid translocase (*Fat* or *CD36*), glucose transporter 4 (*Slc2a4* or *Glut4*), lactate dehydrogenase a (*Ldha*), lactate dehydrogenase b (*Ldhb*), mitochondrial fission protein 1 (*Fis1*), mitofusin 1 (*Mtfn1*), mitofusin 2 (*Mfn2*), nuclear respiratory factor 1 (*Nrf1*), pyruvate dehydrogenase (*Pdh*), peroxisome proliferator-activated receptor gamma coactivator 1 alpha (*Ppargc1a*), peroxisome proliferator-activated receptor alpha (*Ppara*), peroxisome proliferator-activated receptor delta (*Ppard*), peroxisome proliferator-activated receptor gamma (*Pparg*), stearoyl-CoA desaturase (*Scd1*), sterol regulatory element-binding protein (*Srebp1*), TATA box binding protein (*Tbp*), and mitochondrial transcription factor A (*Tfam*).

Gene Abbreviation	**Forward Sequence**	**Reverse Sequence**
Actb	5’-GCCTCACTGTCCACCTTCCA-3'	5’-GGGCCGGACTCATCGTACT-3'
Atp5b	5’-AGGCCCTTTGCCAAGCTT-3'	5’-TTCTCCTTAGATGCAGCAGAGTACA-3'
Bcat2	5’-CGGACCCTTCATTCGTCAGA-3'	5’-CCATAGTTCCCCCCCAACTT-3'
Bckdha	5’-CCAGGGTTGGTGGGATGAG-3'	5’-GGCTTCCATGACCTTCTTTCG-3'
Cebp	5′-GTGTGCACGTCTATGCTAAACCA-3’	5′-GCCGTTAGTGAAGAGTCTCAGTTTG-3’
Cox1	5’-GGTCAACCAGGTGCACTTTT-3'	5’-TGGGGCTCCGATTATTAGTG-3'
Cox5a	5’-GCTGCATCTGTGAAGAGGACAAC-3'	5’-CAGCTTGTAATGGGTTCCACAGT-3'
Cs	5’-TGAGAGGCATGAAGGGACTTGTGT-3'	5’-ATCTGTCCAGTTACCAGCAGCCAA-3'
Cytb	5’-ATATACACGCAAACGGAGCC-3'	5’-TAGGGCCGCGATAATAAATG-3'
Drp1	5’-TGCCTCAGATCGTCGTAGTG-3'	5’-TCTGGTGAAACGTGGACTAGC-3'
Fasn	5’-TTGCTGGCACTACAGAATGC-3'	5’-AACAGCCTCAGAGCGACAAT-3'
Fat(CD36)	5′-TAGTAGAACCGGGCCACGTA-3’	5′-CAGTTCCGATCACAGCCCAT-3’
Fis1	5’-CAAAGAGGAACAGCGGGACT-3'	5’-CAACAGCCCTCGCACATACTT-3'
Gapdh	5’-AGGTCGGTGTGAACGGATTTG-3'	5’-TGTAGACCATGTAGTTGAGGTCA-3'
Slc2a4(Glut4)	5’-GATGAGAAACGGAAGTTGGAGAGA-3'	5’-GCACCACTGCGATGATCAGA-3'
Ldha	5′-GGCTTGTGCCATCAGTATCT-3’	5′-CCCGCCTAAGGTTCTTCATTAT-3′
Ldhb	5’-AGTCTCCCGTGCATCCTCAA-3'	5’-AGGGTGTCCGCACTCTTCCT-3'
Mfn1	5’-CACTGCAATCTTCGGCCAGT-3'	5’-TTCTGGATTCCTGTATGTTGCTTCA-3'
Mfn2	5’-TGATGTGGCCCAACTCCAAG-3'	5’-GTAACATCGATCCCAGGGCTGT-3'
Nd1	5’-CCTTCGACCTGACCTGACAGAAGGA-3'	5’-GATGCTCGGATCCATAGGAA-3'
Nd5	5’-GCTCTACCTCACCATCTCTTGC-3'	5’-TCCAGTATGCTTACCTTGTTACG-3'
Nrf1	5’-ACCCTCAGTCTCACGACTAT-3'	5’-GAACACTCCTCAGACCCTTAAC-3'
Pdh	5’-GAAGGCCCTGCATTCAACTTC-3'	5’-ATAGGGACATCAGCACCAGTGA-3'
Ppargc1a	5’-GACAATCCCGAAGACACTACAG-3'	5’-AGAGAGGAGAGAGAGAGAGAGA-3’
Ppara	5’-CTCGCGTGTGATAAAGC-3'	5’-CGATGCTGTCCTCCTTG-3'
Ppard	5’-GCCTCGGGCTTCCACTAC-3'	5’-AGATCCGATCGCACTTCTCA-3'
Pparg	5′-TTCAGCTCTGGGATGACCTT-3’	5′-CGAAGTTGGTGGGCCAGAAT-3’
Scd1	5′-CATCGCCTGCTCTACCCTTT-3’	5′-GAACTGCGCTTGGAAACCTG-3’
Srebp1	5′-ATCGCAAACAAGCTGACCTG-3’	5′-AGATCCAGGTTTGAGGTGGG-3’
Tbp	5’-GGGATTCAGGAAGACCACATA-3’	5’-CCTCACCAACTGTACCATCAG-3’
Tfam	5’-GAAGGGAATGGGAAAGGTAGAG-3'	5’-ACAGGACATGGAAAGCAGATTA-3'

Supplemental Figure Legends

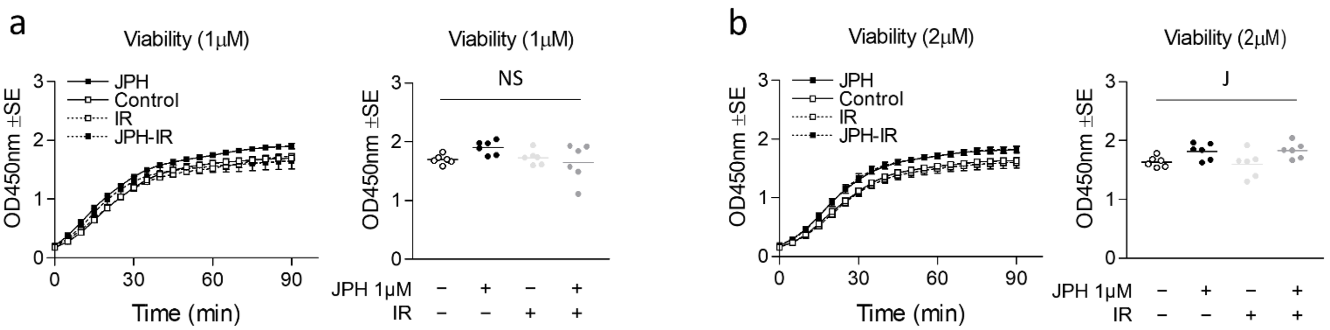

Figure s1 Figure s1 WST-1 cell viability assays. (a) Effect of JPH-203 (JPH) at 1µM (or DMSO 0.1% *v:v*) for 24 hours with and without insulin resistance (IR) on cell viability time trial (left) and endpoint (right). (b) Effect of JPH at 2µM (or DMSO 0.2% *v:v*) for 24 hours with and without IR on cell viability time trial (left) and endpoint (right).

Notes: Two-way and one-way ANOVA with Bonferroni's correction for multiple comparisons were used to assess differences. Dissimilar letters within each graph and above each group indicate p≤0.05 between groups, while J, IR, and I represent main and interaction effects for two-way ANOVA (with NS indicating no significant effect). Measurements were performed using n=3 individual replicates per treatment condition and were repeated across 2 independent experiments with n=6 per group in the final analyses.

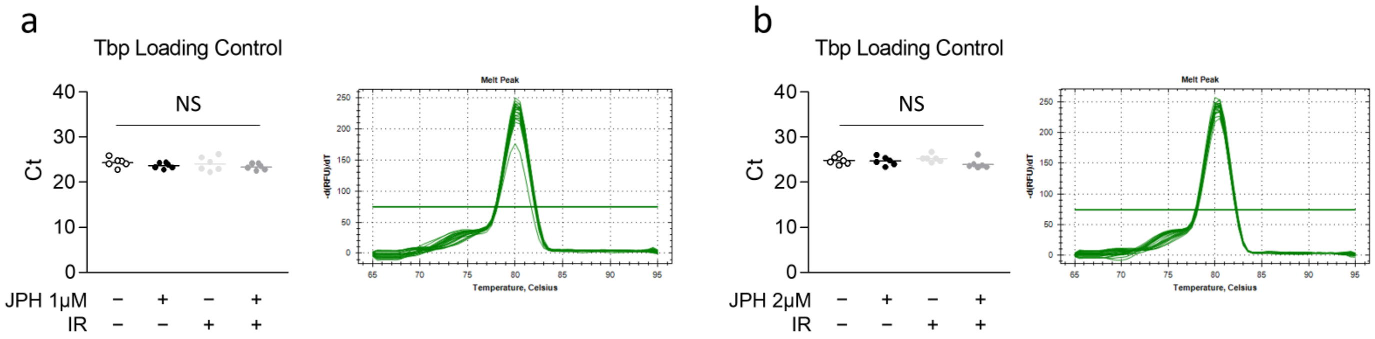

Figure s2 qRT-PCR loading control. (a and b) Effect of JPH-203 (JPH) at (a) 1µM or (b) 2µM for 24 hours with and without insulin resistance (IR) on mRNA expression of tata binding protein (*Tbp*) using 3 replicates per group across 2 independent experiments with n=6 for the final analysis.

Notes: Two-way and one-way ANOVA with Bonferroni's correction for multiple comparisons were used to assess differences in *Tbp* expression. No group differences were observed. NS indicates no significant main or interaction effects.

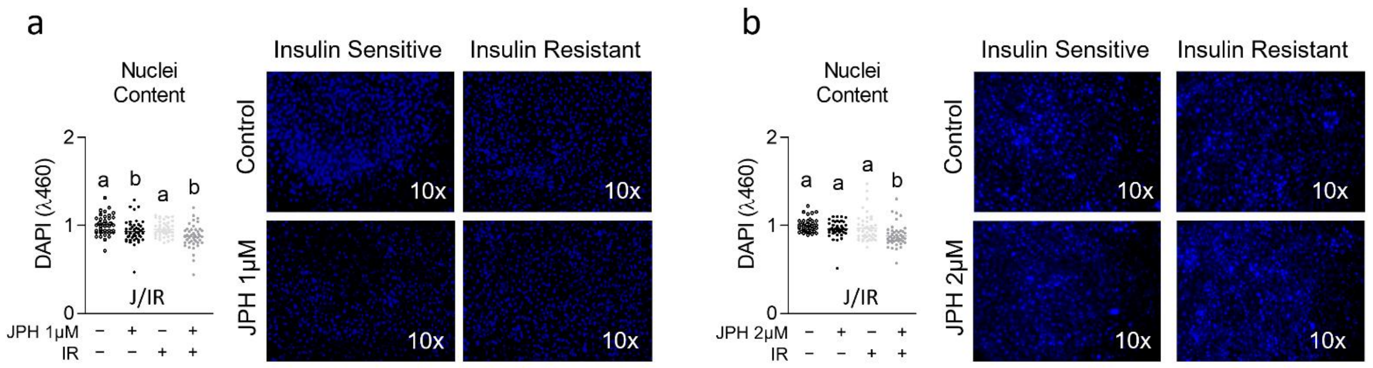

Figure s3 Nuclei content from Seahorse metabolic assays. (a and b) Effect of JPH-203 (JPH) at (a) 1µM or (b) 2µM for 24 hours with and without insulin resistance (IR) on relative nuclei content indicated by DAPI staining.

Notes: Two-way and one-way ANOVA with Bonferroni's correction for multiple comparisons were used to assess differences in DAPI staining. Dissimilar letters indicate p≤0.05 between groups, while J, IR, and I represent main and interaction effects for two-way ANOVA (with NS indicating no significant effect). Measurements were performed using n=23 individual replicates per treatment condition and were repeated across 2 independent experiments with n=46 per group in the final analyses.

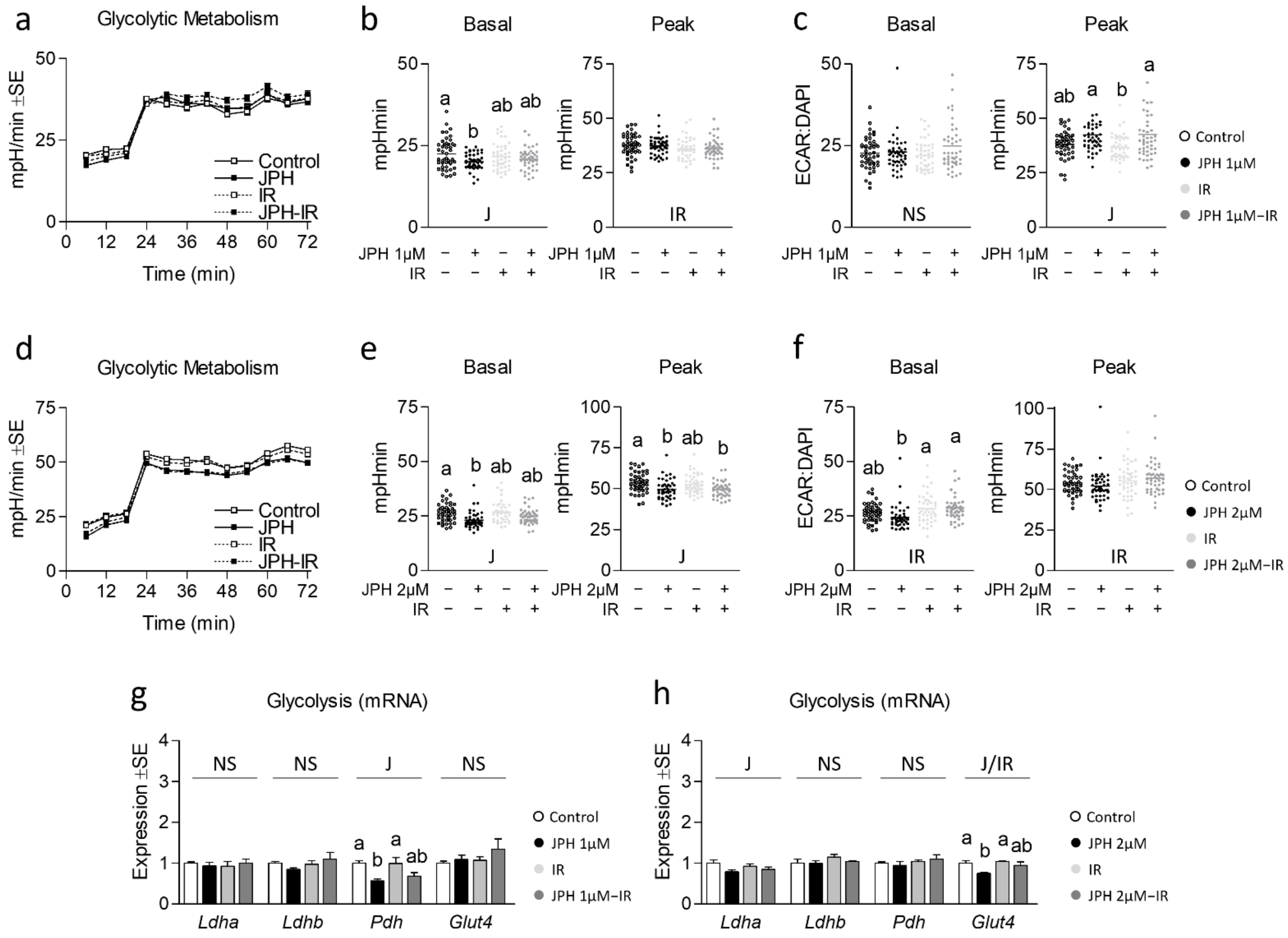

Figure s4 Effect of JPH with and without insulin resistance on glycolytic metabolism and related gene expression. (a) Time course of the effect of JPH-203 (JPH) at 1µM for 24 hours with and without insulin resistance (IR) on mitochondrial metabolism. (b and c) Effect pf JPH with and without IR on basal and peak glycolytic metabolism (b) without and (c) with normalization to nuclei content (Fig s2). (d) Effect of JPH with and without IR on mRNA expression of lactate dehydrogenase a (*Ldha*), lactate dehydrogenase b (*Ldhb*), pyruvate dehydrogenase (*Pdh*), and glucose transporter 4 (*Glut4*).

Notes: Two-way and one-way ANOVA with Bonferroni's correction for multiple comparisons were used to assess differences in metabolism and gene expression following 24-hour treatment. Dissimilar letters indicate p≤0.05 between groups, while J, IR, and I represent main and interaction effects for two-way ANOVA (with NS indicating no significant effect). Metabolic measurements were performed using n=23 individual replicates per treatment condition and were repeated across 2 independent experiments with n=46 per group in the final analyses. No wells responded with negative raw values. Target gene expression was normalized to average tata binding protein (*Tbp*) using 3 replicates per group across 2 independent experiments with n=6 for the final analysis.

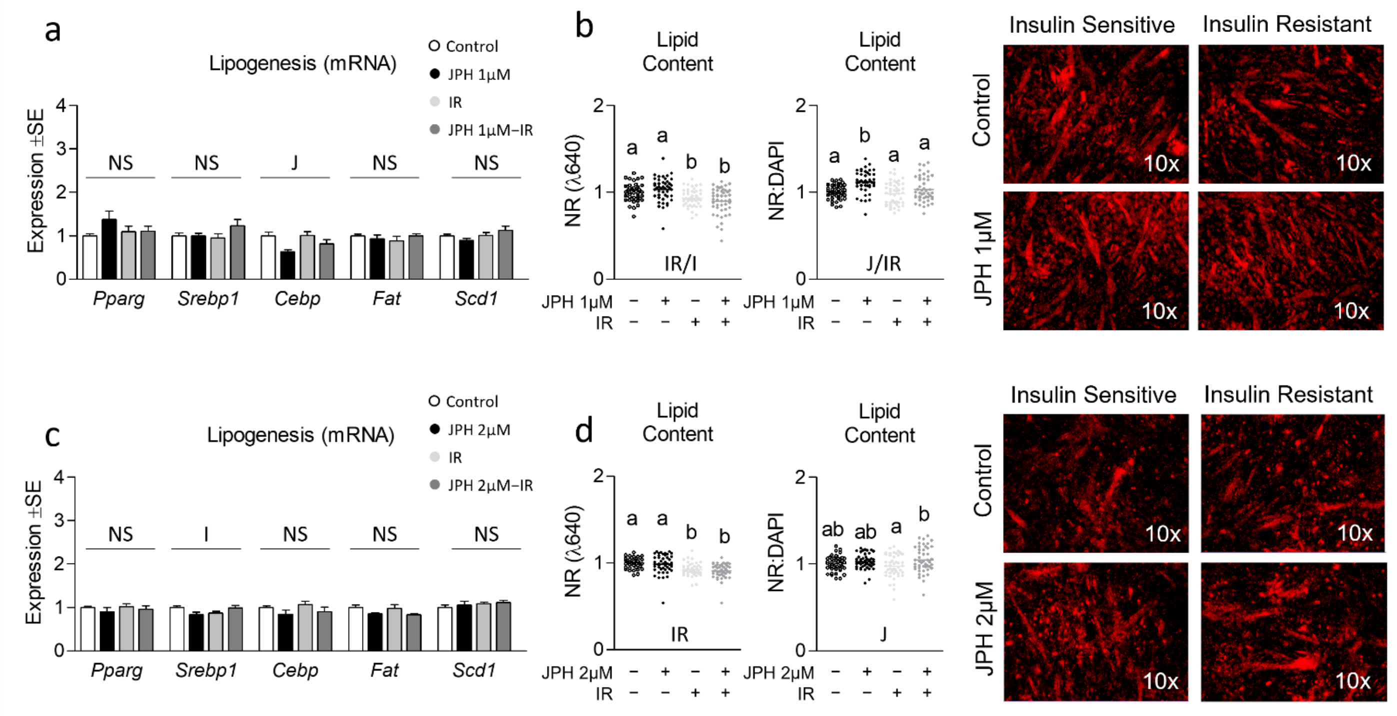

Figure s5 Effect of JPH with and without insulin resistance on lipogenesis and lipid content. (a) Effect of JPH-203 (JPH) at 1µM for 24 hours with and without insulin resistance (IR) on mRNA expression of peroxisome proliferator-activated receptor gamma (*Pparg*), sterol regulatory element-binding protein (*Srebp*), CCAAT/enhancer-binding protein alpha (*Cebpa*), fatty acid translocase (*Fat* or *Cd36*), and stearoyl-CoA desaturase (*Scd1*). (b) Neutral lipid staining (Nile Red) following treatment as described in "a" for 24 hours without (left) and with (right) normalized to nuclei content (supplemental Fig s2) with representative images at right. (c) Effect of JPH at 2µM for 24 hours with and without IR on mRNA expression of *Pparg*, *Srebp*, *Cebpa*, *Fat*, and *Scd1*. (d) Neutral lipid staining following treatment as described in "c" for 24 hours without (left) and with (right) normalized to nuclei content (supplemental Fig s2) with representative images at right.

Notes: Two-way and one-way ANOVA with Bonferroni's correction for multiple comparisons were used to assess differences in lipid content and gene expression following 24-hour treatment. Dissimilar letters indicate p≤0.05 between groups, while J, IR, and I represent main and interaction effects for two-way ANOVA (with NS indicating no significant effect). Lipid staining was performed using n=23 individual replicates per treatment condition and were repeated across 2 independent experiments with n=46 per group in the final analyses using the average of 3 measurements per experiment less background. Images in "b" and "d" of representative individual myotubes were taken using the 10X objective. Target gene expression was normalized to average tata binding protein (*Tbp*) using 3 replicates per group across 2 independent experiments with n=6 for the final analysis.
